# The Significance of the Mediterranean Diet in the Management of Non-Alcoholic Fatty Liver Disease: A Systematic Review

**DOI:** 10.7759/cureus.15618

**Published:** 2021-06-13

**Authors:** Harpreet Gosal, Harsimran Kaur, Hyginus Chakwop Ngassa, Khaled A Elmenawi, Vishwanath Anil, Lubna Mohammed

**Affiliations:** 1 Internal Medicine, California Institute of Behavioral Neurosciences & Psychology, Fairfield, USA; 2 Internal Medicine, Government Medical College, Amritsar, IND; 3 Medicine, California Institute of Behavioral Neurosciences & Psychology, Fairfield, USA; 4 Gastrointestinal Surgery, Faculty of Medicine and Surgery, University of Brescia, Brescia, ITA; 5 Surgery, California Institute of Behavioral Neurosciences & Psychology, Fairfield, USA; 6 Surgery, Cairo University, Cairo, EGY; 7 Internal Medicine/Surgery, California Institute of Behavioral Neurosciences & Psychology, Fairfield, USA

**Keywords:** nonalcoholic fatty liver disease (nafld), mediterranean diet, nash and steatosis, olive oil, plant-based diet

## Abstract

Non-alcoholic fatty liver disease (NAFLD) is the accumulation of intrahepatic fat occurring in the absence of alcohol abuse. The fatty changes in the liver are often the beginning of sequelae of complications, potentially causing steatohepatitis, liver cirrhosis, and hepatocellular carcinoma. The Mediterranean diet is not only a way of eating but is considered a lifestyle and primarily consists of a plant-based diet, with olive oil as the primary lipid. In this study, we reviewed the effectiveness of the Mediterranean diet on NAFLD and its efficacy in disease management. This systematic review follows the Preferred Reporting Items for Systematic Reviews and Meta-Analyses Protocol (PRISMA-P) 2009 guidelines. The PubMed database was used to gather articles, using the following terms individually and in combination, “Mediterranean diet,” “non-alcoholic fatty liver disease,” “insulin resistance,” “metabolic syndrome,” “omega-3-fatty acids.” A quality appraisal was completed to include 14 articles in this systematic review. The studies discuss the correlation between the Mediterranean diet and its role in preventing and treating NAFLD. Olive oil is the major monounsaturated fatty acid, whereas nuts, seeds, and fish consist largely of polyunsaturated fatty acids, both of which are essential components of the Mediterranean diet. The plant-based diet, having sufficient amounts of vegetables, legumes, and fruits, provides its anti-oxidant and anti-inflammatory effects, playing a fundamental role in preventing oxidative stress damage. Olive oil polyphenols increase apoptosis and cell cycle arrest. They also decrease proliferation and angiogenesis, all of which prevent neoplasia. Adapting the Mediterranean lifestyle has shown promising effects in NAFLD, reducing overall mortality and morbidity.

## Introduction and background

The diagnosis of non-alcoholic fatty liver disease (NAFLD) is considered when intrahepatic fat accumulation exceeds 5% in the absence of alcohol abuse [1]. The disease prevalence is estimated to be a quarter of the world population, with a continuing rise [2]. Currently, it is the most common cause of abnormal liver tests in developed countries, with a prevalence of 46% in the United States [3,4]. The occurrence of fatty changes is only the beginning of the potentially life-threatening complications occurring over time, including steatohepatitis, liver cirrhosis, and hepatocellular carcinoma [1]. NAFLD is the leading cause of liver transplantation in men and the second leading cause in women [5]. The manifestations from NAFLD cause sustained liver damage and negatively affected the cardiovascular system, increasing overall mortality, and morbidity [5].

The Mediterranean diet dates back to the traditional dietary patterns from the Mediterranean regions, which are considered a virtual geographic location consisting of numerous cultures [6,7]. The Mediterranean diet has many variations, but its main emphasis has been a plant-based diet, with significant fiber, anti-oxidants, vegetable proteins, polyunsaturated, and monounsaturated fat [8]. It is known as a high-fat diet with olive oil as the principal source, and the fat content ranging from 35% to 45% of the total energy intake [6]. The accumulation of fat occurring due to an imbalance in hepatic lipid metabolism is due to insulin resistance [3]. Hence, insulin resistance is known as the initial step in NAFLD. It is responsible for forming reactive oxygen species (ROS), causing oxidative stress, lipotoxicity, subsequent inflammation, and hepatic damage [3]. The high fruit and vegetable content in the Mediterranean diet has sufficient anti-oxidant and anti-inflammatory properties, counteracting the effects of ROS and the pathogenesis of NAFLD [3]. A similar effect has been observed with olive oil, decreasing inflammation by inhibiting transcription genes [6]. The anti-oxidant effects of the Mediterranean diet seem to share some form of inverse correlation with the underlying pathology of NAFLD.

Research shows that several diets have been tested for NAFLD with various effects. The question remains, is the Mediterranean diet a potential treatment and preventive measure for NAFLD in the near future? It is also the weight loss in response to the diet that significantly affects the disease progression, or is it the ingredients that make the Mediterranean diet that play an essential role [3]? The current treatment recommendations for patients with NAFLD are explored in this article, focusing on how effective the Mediterranean diet is in disease management. Also, we analyze whether adopting the Mediterranean diet early in the steatosis stage of the disease results in any alteration in disease outcome. The fundamental aim of this review is to report the current evidence about the effects of the Mediterranean diet in NAFLD patients and translate this main dietary recommendation to guidelines for clinical practice for disease prevention and treatment.

## Review

This section will discuss how articles were gathered and reviewed for this systematic review. The review analyzes the significance of monosaturated and polyunsaturated fats, the anti-inflammatory effects of the Mediterranean diet, the benefits of limiting sodium, and how these factors alter the outcome of NAFLD.

Methods

The protocol of this systematic review follows the Preferred Reporting Items for Systematic Reviews and Meta-Analyses Protocol (PRISMA-P) 2009 guideline. The electronic database PubMed was searched for articles. The keywords and medical subject headings (MeSH) terms were used individually and in combination to identify relevant articles. The keywords and MeSH terms included in the search strategy were “Mediterranean diet,” “non-alcoholic fatty liver disease,” “insulin resistance,” “metabolic syndrome,” and “omega-3-fatty acids.” A total of 1108 publications were checked for duplications and assessed for relevance to the research topic. The articles were then screened using the inclusion and exclusion criteria to identify the eligibility of the studies. Table 1 and Table 2 summarize the search strategy using MeSH terms and keywords.

**Table 1:** Search strategy with MeSH terms MeSH: medical subject headings; NAFLD: non-alcoholic fatty liver disease

Keyword	Database	Before inclusion/exclusion	After inclusion/exclusion
Insulin resistance [MeSH] AND fatty steatosis [MeSH]	PubMed	1056	136
Mediterranean diet [MeSH]	PubMed	341	79
NAFLD [MeSH] AND omega-3-fatty acids [MeSH]	PubMed	101	34
NAFLD [MeSH] AND Mediterranean diet [MeSH]	PubMed	4	4
Insulin resistance [MeSH] AND Mediterranean diet [MeSH]	PubMed	6	0

**Table 2:** Search strategy with keywords

Keyword	Database	Number of results
Non-alcoholic fatty liver disease	PubMed	20,220
Mediterranean diet	PubMed	7903
Insulin resistance and Mediterranean diet	PubMed	622
Metabolic syndrome and Mediterranean diet	PubMed	551
NAFLD and omega-3-fatty acids	PubMed	307
NAFLD and Mediterranean diet	PubMed	154

Inclusion and Exclusion Criteria

The studies conducted in the last five years (2016-2021), studies based on human subjects, English literature only, studies comprised a mixture of systematic reviews, observational (case-control, cohort, and cross-sectional studies), meta-analysis, and literature review were included. The animal studies and articles with a publication date more than five years ago were excluded.

Results

**Figure 1:**
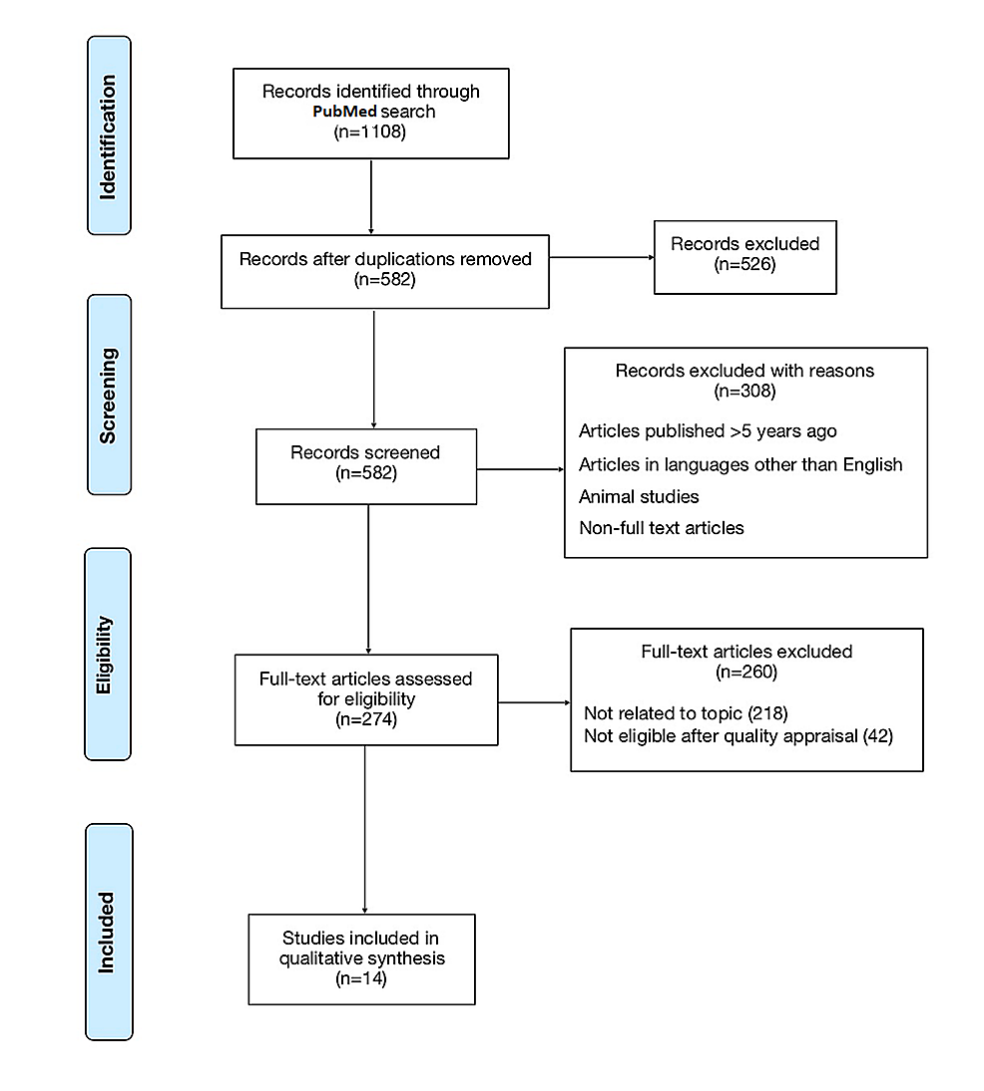
PRISMA flow diagram PRISMA: Preferred Reporting Items for Systematic Review and Meta-Analysis

A total of 1108 publications were identified from PubMed, from which 526 duplicates were removed. Filters were applied based on inclusion criteria (full-text studies in English, last five years, on humans, all types of reviews), yielding 274 articles. Each article was manually reviewed for its content, and articles irrelevant to the study topic were excluded. The eligibility of the remaining articles was checked using the PRISMA checklist, Joanna Briggs Institute (JBI) checklist, New Castle Ottawa Tool (developed as a collaboration between the University of Newcastle, Australia, and the University of Ottawa, Canada), and a measurement tool to assess systematic reviews (AMSTAR) checklist. A total of 14 studies were ultimately selected and included in the qualitative synthesis. Figure 1 below shows the PRISMA flowchart diagram of literature retrieval.

Discussion

**Figure 2:**
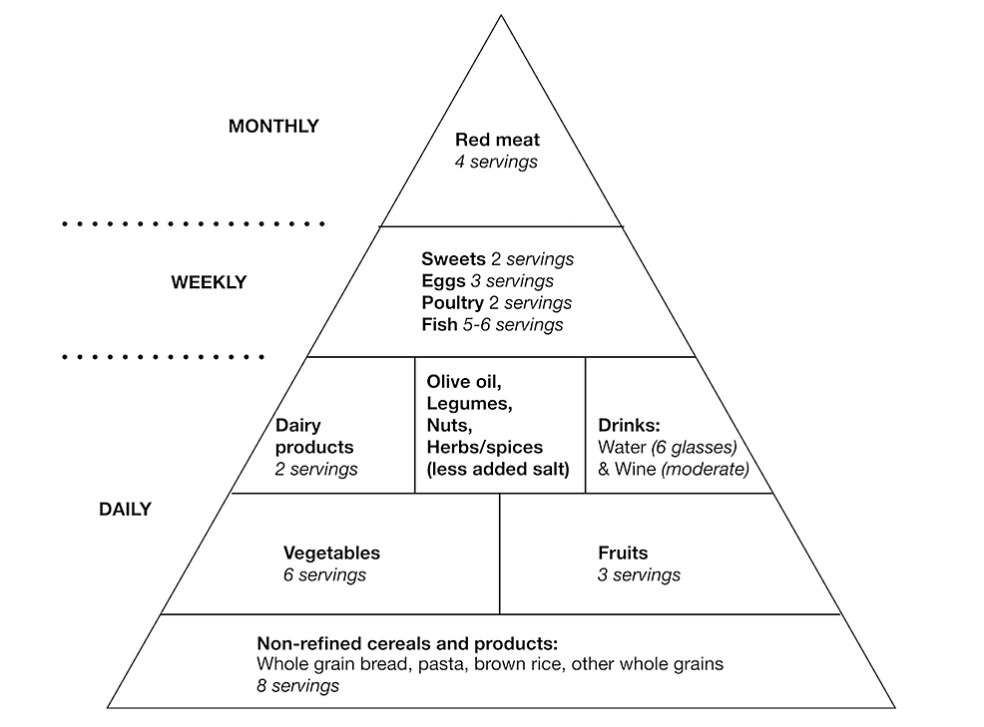
Mediterranean diet pyramid

Figure 2 below shows the Mediterranean diet food pyramid with daily, weekly, and monthly consumptions [5].

Daily

The daily servings consist of high consumption of non-refined cereals and grain products, and vegetables and fruits, along with pulses, legumes, and nuts [9]. Olive oil is the main lipid and considered an essential part of the Mediterranean diet. Also, salt intake is limited by replacing herbs and spices at the table [7]. Besides, adequate water intake, approximately six glasses, is ideal, along with wine in moderation. The consumption of red wine with meals dates back to the traditional Mediterranean diet of the European region [7].

Weekly

Eggs, poultry, and fish are limited to weekly intake, with fish having the highest recommended servings of five or six. Sweets are often limited to a maximum of two servings in the week.

Monthly

The Mediterranean diet also focuses on reducing red meat consumption, with only four servings per month [10]. As mentioned above, the main focus of fat is switched over to olive oil. Hence, red meat intake is significantly decreased.

Olive oil and the role of monounsaturated fat

Olive oil is the most representative group of the Mediterranean diet, and oleic acid, a monounsaturated fatty acid (MUFA), is a major component of this essential dietary fat [5]. MUFAs have numerous beneficial effects, including healthy blood lipid profiles, lowering blood pressure, and improving insulin sensitivity which is an essential and first step in preventing NAFLD [3]. Monounsaturated and polyunsaturated fatty acid (PUFA) diets reduce fasting plasma triacylglycerol and very-low-density lipoprotein (VLDL)-cholesterol concentration on average 19% and 22%, respectively [11]. They also cause an increase in high-density lipoprotein (HDL)-cholesterol levels, which is highly beneficial as current research emphasizes higher HDL levels as preventive measures for long-term diseases [12]. The studies indicate a cardiovascular risk reduction and an all-case mortality reduction linked to the benefits of olive oil [13]. The long-standing conditions show a close relationship to NAFLD prevalence, for example, with over 50% of cases occurring in type 2 diabetes mellitus (T2DM) [14]. The large intake of MUFAs as a substitute for trans fatty acids is believed to reduce the risk of T2DM in high-risk patients by up to 83% over a median of 4.4 years [14]. NAFLD is limited to insulin resistance and includes a spectrum of liver conditions associated with intrahepatic fat accumulation and further complications. For example, a quarter of NAFLD patients progress to non-alcoholic steatohepatitis, which is also associated with a higher risk of cardiovascular disease and mortality [14]. In conclusion, a healthy lipid panel is essential in eliminating countless long-standing health concerns. Monounsaturated fatty acids, driven from olive oil, seem to play an outstanding role in preventive medicine.

Nuts and seeds and the role of polyunsaturated fatty acid

Asides from oil olive as the main source of MUFA in the Mediterranean diet, high quantities of PUFAs in nuts, seeds, and fish also play a valuable role [15]. The most important PUFA for NAFLD is long-chain n-3 fatty acids, which include eicosapentaenoic, EPA, and docosahexaenoic, DHA [15]. These prevent liver adipogenesis by modifying transcription and suppressing peroxisome proliferator-activated receptor α and sterol regulatory element-binding protein-1 genes [16]. The main role of these genes is the regulation of hepatic fatty acid oxidation and synthesis, respectively [16]. Additionally, n-3 fatty acids suppress the direct uptake of fat into hepatocytes and modulate the expression of particular miRNAs [16,17]. The well-established outcome of PUFA and NAFLD by research is the prevention of hepatic steatosis, resulting in a beneficial role in disease management [18].

Effects of olive oil and a plant-based diet on inflammatory-related mechanisms

A bulk of the Mediterranean diet comprises plant-based foods, with a focus on plant-based protein sources. This includes legumes, moderate consumption of fish, and a limited amount of red meat [13]. Vegetables, fruits, and legumes are high in anti-oxidants and anti-inflammatory, playing a fundamental role in preventing oxidative stress damage and cytokine release [13]. From these food sources, legumes have been identified as the most predictive component for lowering mortality [13]. Vegetables and fruits are equally important and consist of polyphenols, which are important bioactive compounds that increase fatty acid oxidation [7]. Polyphenols promote fatty acid beta-oxidation and exert their anti-inflammatory role by inhibiting NF-κB directly or via peroxisome proliferator-activated receptors (PPARs), and chronic inflammatory pathways such as prostaglandin E2 (PGE2) and cyclooxygenase-2 (COX-2) [15]. The recent literature has also shown a relationship between the ability of polyphenols to module the immune system by altering the role of WBCs and inflammatory cytokines that participate in the immunological defense [19]. Hence, the Mediterranean diet has been shown to positively influence inflammatory biomarkers, including adhesion molecules and cytokines, in presenting hepatic fat accumulation and ensuring limited blood levels of transaminase, triglycerides, cholesterol, and insulin resistance [20]. In conclusion, olive oil and plant-based diets, which are rich in polyphenols, have shown to have a positive impact on the human body’s defense mechanism.

Anti-cancer mechanism from olive oil polyphenols

NAFLD is associated with an increased risk of numerous cancers, with a well-established relationship between NAFLD and hepatocellular cancer [21]. However, research has shown that NAFLD is associated with an increased risk for prostate and colon cancer [22]. When comparing the Mediterranean region with other populations, cancer incidence is drastically lower [22]. The role of olive oil and its associated polyphenols, especially oleuropein hydroxytyrosol, is an important component of the underlying anti-cancer mechanism [23]. Polyphenols increase apoptosis and cell cycle arrest while decreasing proliferation and angiogenesis [22]. The role of apoptosis is indicative of the increase in cell death domains, such as caspase-3,-7, p53, and Bax and Bak [22]. At the same time, suppression of neovascularization by olive oil polyphenols has resulted in a decrease in growth factors such as VEGF and TGFalpha-induced migration of hepatocellular carcinoma cells [21]. These mechanisms drastically inhibited the underlying pathology and worked in preventing malignancy.

Limiting sodium

The Mediterranean diet aims to replace salt at the table with herbs and spices, ensuring favor yet adequate blood pressure control. The relationship between high sodium levels and blood pressure is well established, but the direct effect on NAFLD is controversial. The current World Health Organization (WHO) guidelines indicate a sodium intake of <2000 mg/dl [24]. Many epidemiological studies have proven to show a positive correlation between sodium intake and body weight, independent of energy or sugar intake [24]. The literature has established a relationship between increase sodium intake and triglyceride levels, indicating a potential risk factor for NAFLD [24]. The recommendation of a low-sodium diet has been beneficial in lowering overall mortality and morbidity, with hypertension being an underlying cause of many long-standing conditions [25].

Table 3 summarizes experimental and observational studies that were reviewed, and the study group analyzed along with the findings. These studies involved smaller populations but illustrated a positive correlation between NAFLD and the Mediterranean diet in numerous ways. This includes the reduction of intrahepatic fat accumulation and steatosis in relation to consuming the Mediterranean diet and an overall positive impact on NAFLD score.

**Table 3:** Observational and experimental studies illustrating the relationship between NAFLD and the Mediterranean diet NAFLD: non-alcoholic fatty liver disease

References	Study type	Study characteristics	Method	Outcome
Biolato et al., 2019 [18]	Crossover	90 Caucasian patients, 90% male, median age 43 years, body mass index 309 with NAFLD (confirmed by biopsy)	Participants underwent 16 weeks of the Mediterranean diet, 16 weeks of a free wash-out, and 16 weeks of the low-fat diet	Mediterranean diet can improve intestinal permeability in patients with NAFLD, hence is effective in treating visceral obesity and elevated serum transaminase
Mirizzi et al., 2019 [9]	Cross-sectional study	136 participants	Diagnosis of NAFLD was determined using vibration-controlled elastography on a FibroScan and categorized as either NAFLD absent (<215 dB), mild (215-250 dB), and severe (>300 dB)	A greater number of participants that were overweight, obese, and with a higher waist circumference fell into the Severe NAFLD category
Franco et al., 2020 [2]	Observational study: randomized clinical trial	144 moderate or severe NAFLD patients	Participants were assigned six interventions arms during three months, which included control diet, low glycemic index Mediterranean diet, aerobic activity program, combined activity program, low glycemic index Mediterranean diet plus aerobic activity program, or low glycemic index Mediterranean diet plus combined activity program	NAFLD score significantly reduced after 45 days of treatment in every working arm except for the control diet group
Yaskolka et al., 2020 [1]	Observational study: randomized clinical trial	294 participants with abdominal obesity/dyslipidemia	Participants assigned into one of the three groups: healthy dietary guidelines, Mediterranean diet, or green-Mediterranean weight-loss diet, all accompanied by physical activity	NAFLD prevalence declined with the Mediterranean diet, and after 18 months, both Mediterranean groups had significantly higher total plasma polyphenols. Also, intra-hepatic fraction percentage loss was seen with Mankai and walnuts intake and decreased red/processed meat consumption
Campanella et al., 2020 [8]	Observational study: randomized clinical trial	556 participants	Participants were assigned to one of three diets (Mediterranean diet, low glycemic Mediterranean diet, low glycemic index Mediterranean diet) or the control group. Anthropometric variables were noted for each participant, and the outcome of NAFLD was observed	Low glycemic index Mediterranean diet is more effective than other diets in reducing elevated fasting remnant cholesterol
Baratta et al., 2020 [4]	Cohort study	238 participants	Liver ultrasound and Mediterranean-diet questionnaire was done, along with serum sNox2-dp activity, and serum lipopolysaccharide levels measured for each participant	Out of 238 participants, 193 (81.1%) had liver steatosis, with results suggesting that the Med-Diet improves redox status and can be a beneficial approach in NAFLD

Table 4 summarises the current literature reviews that were used in this study to conclude further and establish the effects of the Mediterranean diet on NAFLD. These studies analyzed the risk factors that were influenced by the Mediterranean diet, lowering the outcomes of NAFLD.

**Table 4:** Literature reviews illustrating the relationship between NAFLD and the Mediterranean diet EASL: European Association for the Study of the Liver; EASD: European Association for the Study of Diabetes; EASO: European Association for the Study of Obesity; NAFLD: non-alcoholic fatty liver disease

References	Study type	Purpose of study	Relevant results/conclusions
Hsu et al., 2017 [3]	Traditional review	Analysis of current treatment and dietary approaches for NAFLD	NAFLD patients should follow a low-sodium and low-low-fructose diet, as the Mediterranean diet has shown to be effective in improving hepatic steatosis
Abenavoli et al., 2017 [7]	Traditional review	Re-evaluate the treatment options for NAFLD and primarily focus on the Mediterranean diet as a potential upcoming first-line treatment option.	Adherence to the traditional Mediterranean diet, rich in antioxidants and polyphenols, is a potential new approach in the treatment and prevention of NAFLD
George et al., 2018 [13]	Traditional review	Review the current evidence for the effects of dietary intake in adults with NAFLD and translate these dietary recommendations to guidelines for clinical practice	Implement of the different components of the Mediterranean diet are likely to reduce the onset and progression of NAFLD
Akhlaghi et al., 2020 [17]	Systematic review and meta-analysis	Review the literature on the benefits of the Mediterranean diet for NAFLD	Adherence to the Mediterranean diet is associated with a reduced risk of overall mortality
Abenavoli et al., 2019 [20]	Traditional review	Evaluate the effects of the Mediterranean dietâ€™s anti-oxidant intake on NAFLD.	The Mediterranean diet is suggested in patients with NAFLD patients as an appropriate approach to prevent its onset and development of further disease complications
Plaz Torres et al., 2019 [10]	Traditional review	Overlooks the benefits of the Mediterranean diet in NAFLD patients and analysis the liver outcome	EASL/EASD/EASO NAFLD guidelines recommend diet and physical activity as the best treatment for steatosis
Mirabelli et al., 2020 [14]	Traditional review	Review current evidence about the role of MedDiet and its effects on insulin resistance-related diseases	The MedDiet provides protection from insulin resistance-related diseases such as obesity, type 2 diabetes mellitus, and NAFLD
Margină et al., 2020 [15]	Traditional review	Review the different cellular pathways that are affected by our everyday dietary habits and look into the next steps in addressing these parameters	Diet plays a major role in regulating many cellular pathways, with certain foods components influencing the development of certain diseases

Limitations

This review only includes studies in the English language. As a result, we may have missed studies published in other proficiencies, such as languages dominant in the Mediterranean regions, limiting the definition of the traditional Mediterranean diet. A publication date within the last five years may also cause a similar limitation. The studies prior to 2016 are excluded, while some may have provided valuable information regarding past dietary measures and established relationships to NAFLD. Most of the studies in this review consist of relatively small sample sizes, which potentially limit the efficacy of the results published. Another limitation is the potential confounders that exist among the studies included in this systematic review. This allows for bias, as confounding factors within studies can alter the overall outcome. Lastly, studies use different methods for measuring NAFLD severity, but the definition of Mediterranean diet varied; hence the intervention amongst the studies may not have been consistent.

## Conclusions

This systematic review outlines the significance of the Mediterranean diet in the management of NAFLD and identifies what has been currently studied, and essentially what needs to be focused on in the future to strengthen this relationship. In this review, observational studies, cross-sectional, crossover, and literature reviews were analyzed, indicating a visible link between the two variables. The Mediterranean diet is a regime and a type of lifestyle that has been shown to positively enhancing well-being. It provides a significant number of advantages, being complex in MUFAs, PUFAs, and polyphenols, indicating its anti-inflammatory and anti-cancer mechanisms. After reviewing the literature, the Mediterranean diet can be considered a major step forward in NAFLD management. It promotes weight loss by replacing saturated fats and carbohydrates and plays a direct role in eliminating NAFLD pathology. Adapting the Mediterranean diet in the early stages may result in alterations in disease outcome, but more studies are required to confirm this correlation. In the near future, along with more extensive studies, we may potentially be able to conclude the Mediterranean diet as the first-line preventive and treatment option for NAFLD. 
